# Cultivating Cultural Sensitivity for Prospective Geriatric Professionals through Study Abroad in Japan

**DOI:** 10.1093/geroni/igaf122.3909

**Published:** 2025-12-31

**Authors:** Aya Yoshikawa, Kenshi Nishino, George King

**Affiliations:** Texas Woman’s University, Texas, United States; Furate Medical and Welfare Corporations, Kitakyushu, Fukuoka, Japan; Texas Woman’s University, Denton, Texas, United States

## Abstract

Studying abroad provides a unique experiential learning opportunity to develop students’ cultural sensitivity, critical thinking, problem-solving, and collaboration skills, which are vital for improving the health and well-being of increasingly diverse older adults. In May 2025, 15 undergraduate students from nursing and social work took part in a three-week study abroad course in Japan, one of the fastest-aging societies in the world. During this course, students visited a geriatric care organization in Kitakyushu that provides clinical care and community support for older adults. This learning environment allowed students to investigate various determinants of health and techniques for promoting health among older adults. Students gained greater understanding of aging issues by interacting with older adults, clinicians, the city mayor, city officials, and representatives of aging agencies. Students also engaged in hands-on therapeutic sessions with older adults and an intergenerational service-learning event with a university student organization. Through reflection journal exercises, students recognized similarities and differences in population aging between Japan and the United States and discussed how culture influences healthcare, as well as how to apply their course experience and knowledge to their future professions. Toward the end of the course, students collaborated to create group presentations on risk factors for pre-assigned chronic illnesses (i.e., dementia, cardiovascular disease, age-related joint and bone disorders) and recommended prevention/management strategies from both Japanese and American perspectives. The challenges of course design and delivery, as well as changes in students’ perspectives on public health and aging issues and healthcare over the course, will be highlighted.

